# Molecular evolution of a-kinase anchoring protein (AKAP)-7: implications in comparative PKA compartmentalization

**DOI:** 10.1186/1471-2148-12-125

**Published:** 2012-07-26

**Authors:** Keven R Johnson, Jessie Nicodemus-Johnson, Graeme K Carnegie, Robert S Danziger

**Affiliations:** 1 Department of Medicine, University of Illinois at Chicago, Chicago, IL, USA; 2 Department of Human Genetics, University of Chicago, Chicago, IL, USA; 3 Department of Pharmacology, University of Illinois at Chicago, Chicago, IL, USA; 4 Jesse Brown VA Medical Center, Chicago, IL, USA; 5 Department of Cardiology, University of Illinois at Chicago, 840S. Wood Street, Chicago, IL 60612, USA

**Keywords:** A-Kinase anchoring protein, Protein evolution, PKA regulatory subunit, PKA compartmentalization, AKAP15/18

## Abstract

**Background:**

A-Kinase Anchoring Proteins (AKAPs) are molecular scaffolding proteins mediating the assembly of multi-protein complexes containing cAMP-dependent protein kinase A (PKA), directing the kinase in discrete subcellular locations. Splice variants from the AKAP7 gene (AKAP15/18) are vital components of neuronal and cardiac phosphatase complexes, ion channels, cardiac Ca^2+^ handling and renal water transport.

**Results:**

Shown in evolutionary analyses, the formation of the AKAP7-RI/RII binding domain (required for AKAP/PKA-R interaction) corresponds to vertebrate-specific gene duplication events in the PKA-RI/RII subunits. Species analyses of AKAP7 splice variants shows the ancestral AKAP7 splice variant is AKAP7α, while the ancestral long form AKAP7 splice variant is AKAP7γ. Multi-species AKAP7 gene alignments, show the recent formation of AKAP7δ occurs with the loss of native AKAP7γ in rats and basal primates. AKAP7 gene alignments and two dimensional Western analyses indicate that AKAP7γ is produced from an internal translation-start site that is present in the AKAP7δ cDNA of mice and humans but absent in rats. Immunofluorescence analysis of AKAP7 protein localization in both rat and mouse heart suggests AKAP7γ replaces AKAP7δ at the cardiac sarcoplasmic reticulum in species other than rat. DNA sequencing identified Human AKAP7δ insertion-deletions (indels) that promote the production of AKAP7γ instead of AKAP7δ.

**Conclusions:**

This AKAP7 molecular evolution study shows that these vital scaffolding proteins developed in ancestral vertebrates and that independent mutations in the AKAP7 genes of rodents and early primates has resulted in the recent formation of AKAP7δ, a splice variant of likely lesser importance in humans than currently described.

## Background

cAMP-dependent protein kinase A (PKA) is a broad-specificity serine/threonine protein kinase that is ubiquitously distributed among tissue types, and identified in organisms from fungi to humans. Inside cells, PKA exists as a tetrameric protein composed of two catalytic subunits and a regulatory subunit dimer. The PKA regulatory subunit (PKA-R) functions to maintain kinase inactivity in the absence of cAMP and form protein-protein interactions that compartmentalize the kinase. The selective sequestration or compartmentalization of intracellular PKA has been supported by several studies showing that PKA is concentrated in discrete intracellular locales by A-kinase anchoring proteins, or AKAPs [1-4]. AKAPs bind to the regulatory (i.e., PKA-RI, PKA-RII or both) subunit dimer of the tetrameric PKA holoenzyme at an antiparallel four-helix domain termed the dimerization and docking (D/D) domain [5-7]. The AKAP-PKA-R interaction functions to sequester PKA in the vicinity of its phosphorylation substrates [2,8,9] and this selective concentration of PKA activity in part, explains how multiple diverse extracellular stimuli can result in highly specific and discrete PKA-mediated phosphorylation events [10,11].

Originally named according to their apparent molecular weight, AKAPs are now designated by gene number (i.e., AKAP15/18 splice variants are referred to as AKAP7 splice variants). Currently there are 14 annotated AKAPs (AKAP1 to AKAP14). The PKA-regulatory subunit binding domain of AKAPs is the most recognizable element among the otherwise structurally diverse protein family. AKAPs are classified according to binding specificity for the PKA regulatory subunits; RI-, RII- or both RI and RII PKA regulatory domains (dual specific) [12]. The primary structure (i.e., amino acid sequence) of the PKARI/RII-binding domain is highly variable among AKAPs, though the secondary structure, which forms an amphipathic helix, contains several conserved hydrophobic residues that determine PKA-R-subunit specificity and binding affinity [13-15]. AKAPs also contain unique localization signal(s) which are responsible for AKAP targeting to sub-cellular compartments, and in many cases, AKAPs also contain enzyme-binding sites that enable the formation of multi-protein signaling complexes [16,17]. Various AKAP targeting domains have been shown to interact with G-protein coupled receptors (GPCRs; [18]), adenylyl cyclases [19], Exchange protein activated by cAMP (EPAC; [20]), and phosphodiesterases (PDEs) [21,22]. Hence, AKAPs function as signal processing hubs specifically linking upstream activation signals to downstream effector proteins.

AKAPs have been shown to regulate neuronal ion channels [23,24], memory and cognition [25-27], reproduction function [22,28], as well as renal [29] and cardiac function [11,30-35]. One AKAP that has been identified in many tissues and a variety of cell signaling pathways is AKAP7 (also known as AKAP15/18). Splice variants of the AKAP7 gene (AKAP7α, β, δ, and γ) are dual-specificity AKAPs that have been extensively studied in central nervous system (CNS), striated muscle ion channel regulation [2,36,37], cardiac calcium cycling [38], and renal aquaporin transport [29].

AKAP7α, the first AKAP7 splice variant identified, is a membrane bound protein containing a lipid modification domain in the amino terminus [39]. AKAP7α binds to L-type Ca^2+^ channels where it regulates the PKA-dependent phosphorylation of the ion channel [40]. AKAP7α has also been shown to interact with sodium channels in the brain [2], and is required for feedback inhibition of the epithelial Na^+^ channel (ENAC) [41]. AKAP7α-mediated PKA anchoring to ion channels (Ca^2+^ and Na^+^) provides a regulatory mechanism for channel function, whereby anchoring of PKA regulates the phosphorylation state of the ion channel and has a profound effect on channel “open probability” and ion movement [1,40,42,43]. Another extensively studied AKAP7 splice variant is AKAP7δ, which was originally identified in rat kidney [29]. In rat renal principle cells, the APAK7δ splice variant functions to anchor PKA to intracellular vesicles containing the aquaporin water channel and is required for aquaporin fusion to the plasma membrane [29]. Also in rats, AKAP7δ has been identified in the cardiac sarcoplasmic-endoplasmic reticulum ATPase (SERCA) where it regulates PKA phosphorylation of a SERCA negative regulatory protein, phospholamban (PLN) and controls Ca^2+^ re-uptake into the sarcoplasmic reticulum (SR) [38]. AKAP7δ has become a prime example for the pharmacological potential of PKA-AKAP disruptor molecules, particularly in the treatment of human heart failure [44,45]. Much less is known about the AKAP7β and γ splice variants. AKAP7β differs from AKAP7α by the inclusion of a third exon which imparts a unique membrane localization that differs from that found for AKAP7α [46]. AKAP7γ has been cloned from human pancreas [46] and mouse oocytes [47], though the functional significance of this splice variant remains relatively unknown.

Though appreciation for the importance of AKAPs in cell signaling has grown exponentially in recent years, an analysis of the evolution and development of an AKAP remains absent. This study addresses several key questions in the molecular evolution of AKAP7 proteins including; 1) evolution/genesis of the AKAP7 RI/RII binding domain in relation to the PKA-R D/D domains 2) evolution of the AKAP7 gene structure 3) species analyses of AKAP7 splice variants, 4) sequence determination of previously un-reported mouse and human AKAP7δ and 5) evolutionary conservation of key protein regions in short AKAP7 splice variants and rapid change in AKAP7 long form splice variants.

## Results

### Evolution of AKAP7 and PKA-R interaction motifs

**Figure 1:**
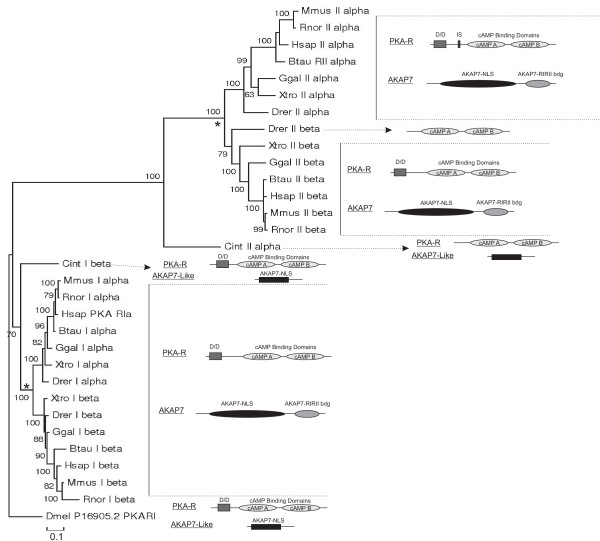
**Co-Evolution of PKA-R and AKAP7 RI/RII binding domains.** A phylogenetic tree was constructed using PKA-R amino acid sequences. Bootstrap values are indicated next to each branch. PKA-R gene duplication events are indicated by *. PKA-R and AKAP7 protein families identified in species composing each clade are shown adjacent to the PKA-R phylogenetic tree. The PKA-R dimerization/docking (D/D) domain is identified in invertebrate and vertebrate species, while the AKAP7 RI/RII binding domain appears in vertebrate species following PKA-R gene duplication events. The PKA-RIIβ subunit in zebrafish lacks the AKAP7 RI/RII binding domain.

**Figure 2:**
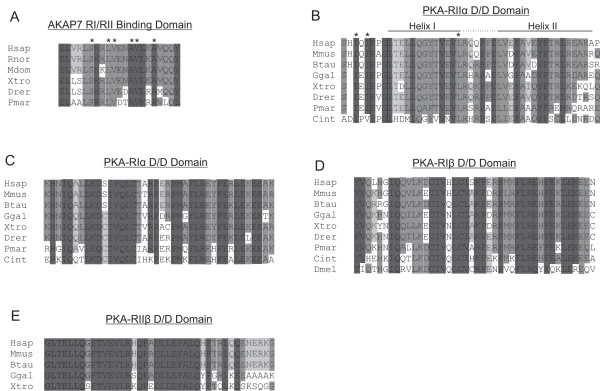
**Amino Acid Alignments of AKAP7 RI/RII binding domain and PKA-R D/D domains.** Protein regions from the AKAP7 RI/RII binding domain (**A**), PKA-RIIα dimerization/Docking domain (D/D; **B**), PKA-RIα D/D (**C**) PKA-RIβ D/D, (**D**), and PKA-RIIβ (**E**) were aligned using Clustal X. (**A**) Residues in the AKAP7 RI/RII binding domain that have been previously identified as components of an amphipathic α-helix necessary for PKA-R subunit interaction are indicated by (*) and residues that are conserved are shaded. In the AKAP7 RI/RII binding domain, α-helix forming residues are conserved in vertebrates from lamprey to humans. Residues in the PKA-RIIα (**B**)**,** PKA-RIα (**C**), PKA-RIβ (**D**), and PKA-RIIβ (**E**) D/D domains forming the docking surface for the AKAP helix which are highly conserved are indicated by shading. Residues in PKA-RIIα that are essential for AKAP binding [48,49] are indicated by (*). The spacer region between helices in PKA-RIIα is indicated by a dotted line.

**Figure 3:**
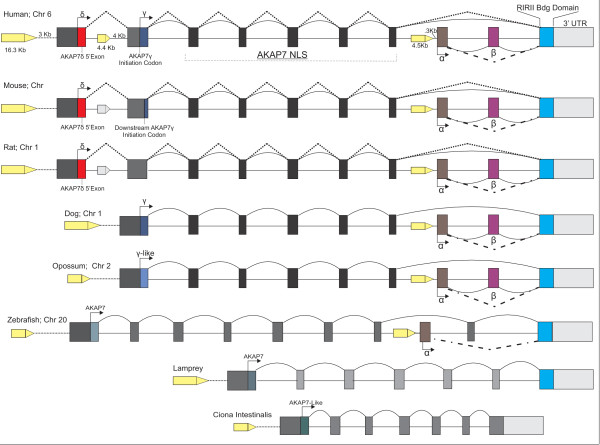
**AKAP7 Gene Evolution.** AKAP7 ESTs from chordate (*Ciona Intestinalis*), basal vertebrate (lamprey), teleost (zebrafish), and mammals (Opossum, Dog, Rat, and Human) were aligned to their respective genomes to determine exon positions and orientation. Coding sequence for the AKAP7 RI/RII binding domain is found in the 3’ terminal exon of AKAP7 splice variants and is identified in the AKAP7 genes from lamprey to humans. The AKAP7α 5’ exon first appears in zebrafish and an ortholog of this exon is identified in the genes of multiple mammals. The AKAP7β-specific exon first appears in the dog AKAP7 gene and is conserved in mammalian genes. An AKAP7 long form splice variant is found in each species, though AKAP7γ orthologs are only identified in species following the divergence of dog. The AKAP7γ 5’ exon is lost in rats, and is concomitant with the incorporation of the AKAP7δ 5’ exon (AKAP7δ exon is identified in the cow AKAP7 gene but no ESTs were detected).

The AKAP7 protein domain required for PKA-R binding (AKAP7 RI/RII binding domain; pfam 10470) is detected in transcripts of species from zebrafish to humans (Figure 1). The 3’ terminal exon encoding the AKAP7 RI/RII binding domain first appears in the AKAP7 gene of lamprey and is conserved throughout vertebrates, placing AKAP7 binding domain genesis at the base of the vertebrate lineage (Figures 2 and 3). Transcripts containing (encoding) the AKAP7RI/RII binding domain were not identified in Drosophila and Ciona, though transcripts containing high sequence identity to the AKAP-7 NLS were identified (termed AKAP7-like; Figure 1). AKAP7-like genes were also identified in *Strongylocentrotus purpuratus* (Additional file 1: Figure S1)*, Chlorella variabilis*, and *Daphnia pulex* (not shown). The resultant putative protein products of these AKAP7-like genes lack the AKAP7RI/ RII binding domain and likely the PKA binding functionality of derived AKAPs.

The PKA-R subunit protein domain necessary for AKAP interaction (the Dimerization/Docking, D/D domain; pfam 02197) is conserved in the PKA-R subunits of insects, urochordates, chordates, and vertebrates (with the exception of Zebrafish PKA-RIIβ; Figures 1 and 2). Specifically, the residues necessary for PKA-R/AKAP7 interaction [5,7,13,15] are conserved in all species examined (Figure 2). Appearance of the AKAP7 RI/RII binding domain corresponds with the vertebrate specific PKA-R gene duplication (Figure 2).

### AKAP7 Splice variant evolution

**Figure 4:**
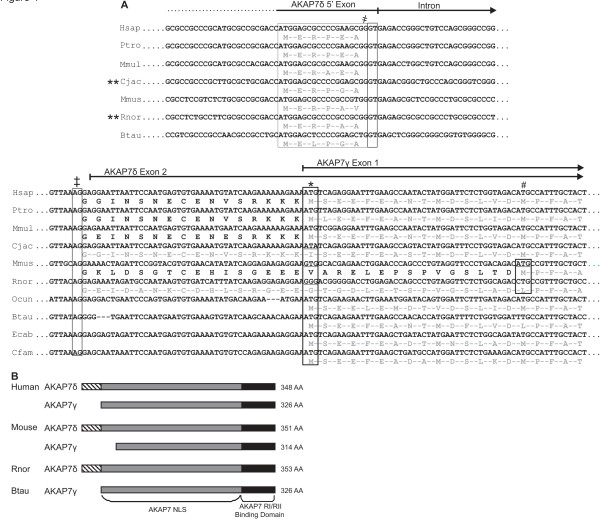
**Multi-species AKAP7 gene alignments.** (**A**) AKAP7 gene sequence and coding regions of AKAP7 long form splice variants δ and γ from several mammals were aligned using Clustal. Translated protein sequence has been added for splice variants with EST or sequencing support from this study. Position of the AKAP7δ 5’ exon and flanking intron are indicated with solid lines and arrows, the 5’ and 3’ splice junctions are denoted with (**҂**). The AKAP7δ 5’ exon is only present in the AKAP7 genes of cow, rat, mouse, and primates. The putative AKAP7γ start codon is indicated with (*), the methionine (M) at this position has mutated to a glycine (G) in rats, valine (V) in mice, and isoleucine (I) in marmoset. AKAP7γ is initiated at a downstream methionine in mice, indicated by (#). This codon is mutated to a leucine (L) in rats. Species where AKAP7δ is the only AKAP7 long form splice variant produced are indicated by (**). (**B**) A protein model was constructed to represent the AKAP7 long form splice variant complement for species shown in (**A**). The ancestral AKAP7γ splice variant is present in cow and human, absent in rat, and truncated in mouse.

AKAP7 gene structure, exon numbers and orientation were determined for multiple species (Figure 3, Additional file 1: Figure S1). AKAP7α coding sequence in the AKAP7 gene and EST transcript evidence first appears in basal teleosts (*Danio rerio*), and is conserved from teleosts to humans (Figure 3, Additional file 1: Figure S1). AKAP7β ESTs are only identified in mammals (pig; *Sus scrofa*; Additional file 1: Figure S1), though the AKAP7β-specific gene coding region is detectable from marsupials to mammals (Figure 3). AKAP7δ is the most recent AKAP7 splice variant, with EST evidence supporting transcript presence in rats through humans. However exon data suggests this splice variant is absent from AKAP7 genes in species below laurasiatherian mammals (Additional file 1: Figure S1, Figure 4). AKAP7δ is formed by the incorporation of a 19-nucleotide exon located between 7- and 10Kb upstream of the putative AKAP7γ 5’ exon from cow to human (Figure 4). The appearance (expression) of the AKAP7δ splice variant coincides with the loss of expression for AKAP7γ in both rat and marmoset (*Calithrix jacchus*) EST databases, as well as the integration of a smaller AKAP7γ 5’ exon in the mouse transcript which results in a truncated AKAP7γ amino terminus unique to mouse (Figure 4).

The sequencing of human and mouse AKAP7δ and alignment with rat AKAP7δ validates the presence of the multiple nucleotide mutations resulting in the truncation of AKAP7γ in mice and loss of AKAP7γ in rats (Additional file 2: Figure S2). Mouse AKAP7δ has a 79% nucleotide sequence identity compared to human AKAP7δ (74 % amino acid identity), and rat AKAP7δ has 78% nucleotide sequence identity to human AKAP7δ. Both rat and mouse AKAP7δ splice variants contain an “RGD” amino acid sequence that is absent in human AKAP7δ (Additional file 2: Figure S2).

### Replacement of AKAP7γ with AKAP7δ indicates similar function

Multiple sequence nucleotide alignments identified mutations in the AKAP7 genes of rat, mouse, and marmoset which result in the loss of (in rat and marmoset) or truncation of (in mouse) the AKAP7γ splice variant and incorporation of the AKAP7δ splice variant ( 4). In the rat AKAP7 gene, the putative AKAP7γ initiation codon contains two nucleotide mutations where “ATG” in the Kozak sequence is mutated to “GGG”, the result of a transition at position 1 of the codon (A → G), and a transversion at position 2 of the codon (T → G) yielding the missense mutation Met → Gly. In the mouse AKAP7 gene, a cognate mutation was also identified in the putative AKAP7γ initiation codon as a single transition at nucleotide position 1 (A → G) replaces the AKAP7γ start codon with “GTG”, resulting in the missense mutation Met → Val. Also identified in the mouse AKAP7 gene, a second mutation downstream of the AKAP7γ initiation codon results in the formation of an internal start site where an “ATG” is formed as the result of a nucleotide transversion (C → A), yielding the amino acid Leu → Met mutation which yields a truncated AKAP7γ splice variant in mouse (Figure 4). The AKAP7γ mutations in the rodent lineage seem to be specific to rats and mice, as another rodent (*Oryctolagus cuniculus*; rabbit) lacks these mutations, and also altogether lacks the AKAP7δ 5’ exon (Figures 3 and 4). The loss of AKAP7γ is also evident in marmoset, as “ATG” is replaced by “ATA”, the result of a transition at position 3 of the initiation codon (G → A) replacing Met with Ile.

**Figure 5:**
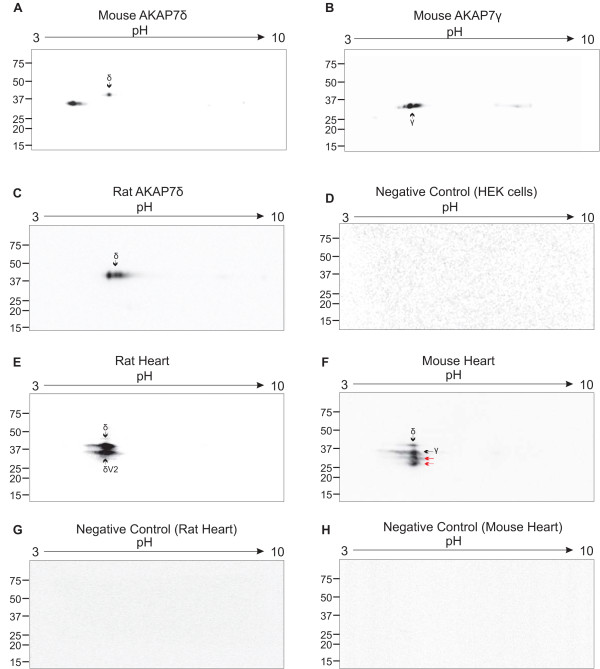
**2D Western Analysis of AKAP7δ and AKAP7γ.** Recombinant mouse AKAP7δ, AKAP7γ, and rat AKAP7δ were analyzed by two-dimensional western blotting. V5-tagged Mouse AKAP7δ (**A**), AKAP7γ (**B**) or rat AKAP7δ (**C**) were detected from transfected HEK293 cell lysates using a V5-HRP antibody. (**D**) Mock-transfected HEK293 cell lysates were probed with V5-HRP as a negative control. (**E**) Rat heart lysate used in AKAP7 immunoprecipitations (using AKAP7 antibody) was probed with protein A-HRP. Two products are identified, rat AKAP7δ, and a variant of rat AKAP7δ identified in this study (rat AKAP7δ variant 1, see Supplemental Information Figure 3). (**F**) Mouse heart lysate subjected to AKAP7 immunoprecipitation followed by detection with protein A-HRP. Mouse AKAP7δ and AKAP7γ are identified in addition to two unknown splice variants (indicated by red arrows). For negative control immunoprecipitations rat heart lysate (**G**) or mouse heart lysate (**H**) was immunoprecipitated with rabbit IgG (pre-immune serum) followed by detection by protein A-HRP. The pH range and molecular weight are indicated at the top and left (respectively) of each blot.

**Figure 6:**
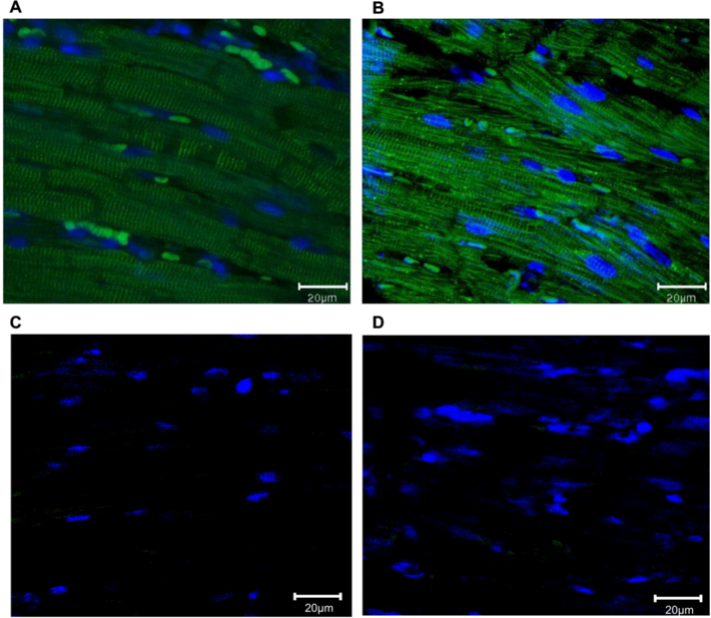
**AKAP7 Localization in rat and mouse heart.** Merged immunofluorescence images of (**A**) adult rat and (**B**) mouse hearts were taken using a confocal microscope. Immunostaining was performed using a rabbit anti-AKAP7 antibody, the 2˚ Ab is Alexa fluor 488 anti-rabbit. Nuclei are stained with Dapi (blue). AKAP7 immunostaining is prominent in the sarcoplasmic reticulum in both rat (expressing AKAP7δ only) and mouse (predominantly AKAP7γ but also AKAP7δ). In the negative controls for rat (**C**) and mouse (**D**) normal rabbit serum (AKAP7 pre-immune serum) was used in place of the AKAP7 primary antibody. Images were taken at 60X magnification.

Two-dimensional western analysis of recombinant rat and mouse AKAP7 long-form splice variants confirms the loss of AKAP7γ in rats, and shows that both AKAP7δ and AKAP7γ are produced from AKAP7δ cDNA from mice (Figure 5). It is also apparent that mouse AKAP7γ is the major product from AKAP7δ cDNA (Figure 5A), which is likely the result of an AKAP7γ internal translational start site that is present in mice, but absent in rats (Figure 4). AKAP7 immunoprecipitations (IPs) identified AKAP7δ and a variant of AKAP7δ (see below) in rat heart, while AKAP7δ, AKAP7γ, and unknown variants were seen from mouse heart IPs (Figure 5). Comparison of rat and mouse AKAP7 cardiac localization by immunofluorescence (Figure 6) shows identical localization compared between rat (AKAP7δ only) and mouse (predominantly AKAP7γ but also AKAP7δ). The pattern of AKAP7 cardiac immunstaining in rat and mouse is consistent with localization to the sarcoplasmic reticulum, as previously reported for AKAP7δ in rat [38].

### Insertion-deletions in human and Rat AKAP7δ yield novel splice variants

**Figure 7:**
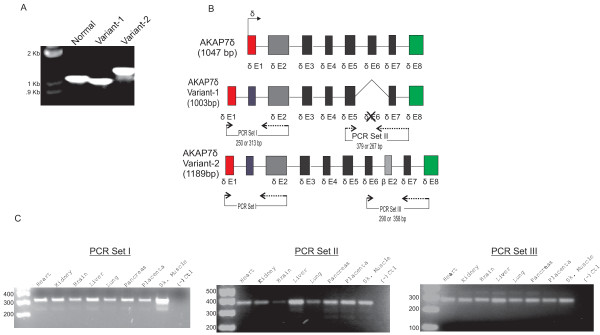
**Insertion-Deletion Variants of Human AKAP7δ.** (**A**) PCR amplification of human AKAP7δ shows two splice variants of different length following agarose gel electrophoresis. (**B**) In AKAP7δ variant-1 an insert sequence is found between AKAP7δ exons-1 and 2 and AKAP7δ exon-6 is deleted. An insert sequence between exons-6 and 7 is identified in AKAP7δ variant-2 in addition to the 5’ insert sequence. (**C**) PCR amplification was used to determine the expression of AKAP7δ variants-1 and 2 in human tissues (relative docking sites for primer pairs are shown in 7B). In PCR set I, the 5’ insert sequence is predominantly present in all tissues relative to the non-insert product (native). In PCR set II, the native form is the major product relative to the AKAP7δ exon-6 deletion found in variant-1. Shown in PCR set III, the 3’ insert sequence found in variant-2 is a minor product in the liver and pancreas relative to the native form.

Sequencing of human AKAP7δ identified splice variants containing nucleotide sequence insertions and deletions (indels). Human AKAP7δ variant-1, contains a seventy five nucleotide insertion between the first and second exons of AKAP7δ (located 5’ to the AKAP7γ start codon; Figure 7), and an exon deletion (AKAP7δ exon 6; Figure 7). AKAP7δ variant-2 contains two insertions; a sixty nine-nucleotide insertion between AKAP7δ exons 1 and 2, and a sixty nine nucleotide insertion between AKAP7δ exons 7 and 8 (Figure 7). The upstream insertion sequence in AKAP7δ variant-1 has 100 % sequence identity to a region in the human AKAP7 gene (located on chromosome 6) between the AKAP7δ 5’ exon and AKAP7δ exon 2. The upstream insertion sequence in variant-2 has 96% sequence identity to the human AKAP7 gene. The downstream insertion sequence in AKAP7δ variant-2 has 100% sequence identity to the AKAP7β-specific sequence. PCR amplification of the human AKAP7δ indel regions show the 5’ insertion is predominant relative to the native form, while the deletion-variant is less prominent than the native transcript form (Figure 7C). The AKAP7δ splice variant containing the AKAP7β exon is a minor product but is identified in most tissues (Figure 7C).

**Figure 8:**
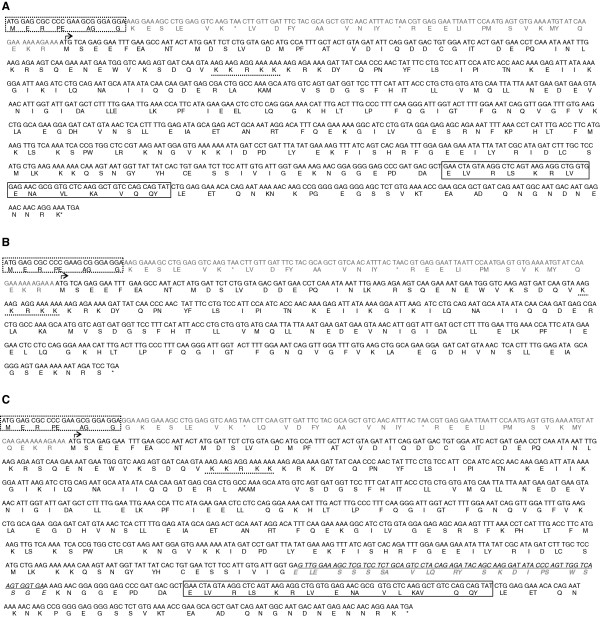
**Open Reading Frame Analysis of Human AKAP7δ insertion-deletion variants.** Human AKAP7δ splice variants were assessed for open reading frames using the NCBI ORF finder. (**A-C**) Coding and protein sequence for the AKAP7δ amino terminus is indicated by a dotted box, reading frame initiation is indicated by an arrow, and the AKAP7 NLS has a dotted underline. The AKAP7 RI/RII binding domain (if present) is identified in a solid box and termination codons are indicated by an (*). (**A**) The 5’ insertion identified in the majority of human tissues contains two termination codons and results in a reading frame shift yielding the AKAP7γ splice variant. (**B**) The 5’ insertion combined with deletion of exon-6 results in a truncated protein with the AKAP7γ amino terminus. (**C**) The 5’ insertion combined with the insertion of the AKAP7β exon yields a protein with the AKAP7γ amino terminus, the AKAP7 NLS, the protein region previously identified only in AKAP7β, and the AKAP7 RI/RII binding domain.

Open reading frame (ORF) analysis of Human AKAP7δ transcript variants-1 and 2 (Figure 8) show that the AKAP7δ 5’ insertions shifts the reading frame downstream to begin initiation at the AKAP7γ coding region (Figure 8A), while the downstream exon deletion seen in AKAP7δ variant-1 results in a truncated protein that contains the AKAP7 nuclear localization signal (NLS), but lacks the AKAP7 RI/RII binding domain (Figure 8B). The open reading frame for AKAP7δ variant-2 begins at the AKAP7γ initiation codon and produces a protein containing the AKAP7 NLS, AKAP7 RI/RII binding domain and the AKAP7β protein region (Figure 8C).

Two exon-deletion transcript variants were also identified in rat AKAP7δ (Additional file 3: Figure S3). AKAP7δ variant-1 contains deletions at exons 3–6, while variant-2 contains a deletion of exon 6. Of these variants, only rat AKAP7δ variant-2 contains the AKAP7 RI/RII binding domain. No indels were detected from sequencing mouse AKAP7δ, however a short (3-exon) AKAP7γ splice variant was detected in mouse ESTs (Acc# BC099487.1), and is marginally expressed in mouse spleen (Additional file 4: Figure S4).

### Conservation in AKAP7 short splice variants

AKAP7 short splice variants (AKAP7α and β) have pronounced sequence conservation across the vertebrate phyla. Nucleotide sequence alignments of the AKAP7 α/β 5’ exon show that this exon is highly conserved from zebrafish to humans (Additional file 5: Figure S5), as the nucleotide sequence homology for this exon ranges from 66 to 98 % identity. Amino acid alignment of the AKAP7α/β amino terminus (Additional file 3: Figure S3) shows this protein region has a sequence homology of 70 to 100% from zebrafish to humans.

AKAP7β is distinguished from AKAP7α by the inclusion of a 69-nucleotide exon between the AKAP7 α/β 5’ exon and the AKAP7 3’ terminal exon. This AKAP7β-specific exon is found in the genomes of several mammals (Figure 3, Additional file 1: Figure S1), and is highly conserved (Additional file 5: Figure S5). Amino acid alignment of the AKAP7β-specific protein region shows sequence homology of 65 to 95% from pigs to human (Additional file 5: Figure S5).

### Tissue Expression of AKAP7 splice variants in rodents and humans

**Figure 9:**
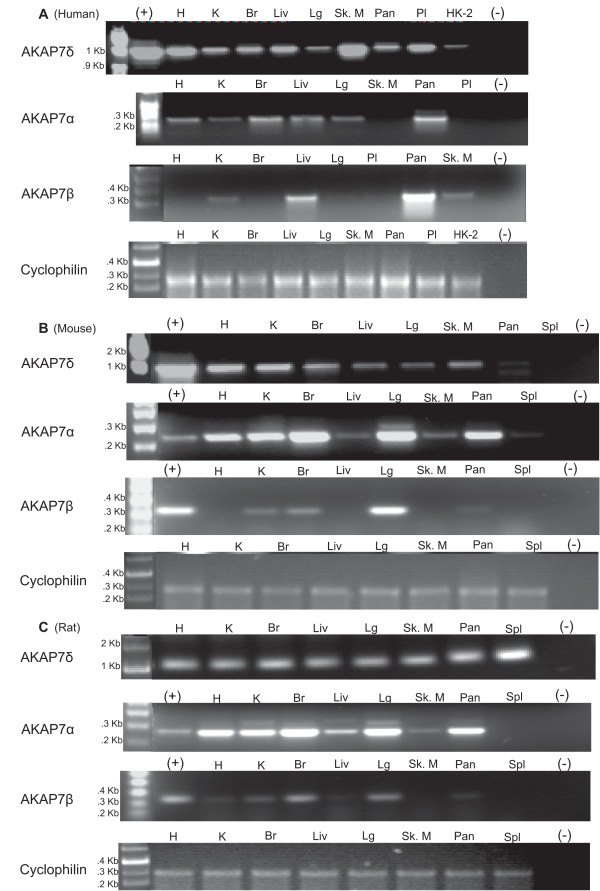
**Tissue Expression of AKAP7 Splice Variants.** PCR amplification and agarose gel electrophoresis of AKAP7δ, α and β splice variants from (**A**) human, (**B**) mouse and (**C**) rat tissue cDNA. AKAP7α tissue expression is conserved among human, mouse, and rat heart, brain, liver and lung. AKAP7β tissue expression is conserved in the kidney, and AKAP7δ is ubiquitously expressed in humans, mice and rats.

AKAP7δ is ubiquitously expressed in human, mouse and rat tissues (Figure 9). AKAP7α expression is expressed in several rodent and human tissues, with conserved expression seen between rat, mouse and human heart, kidney, brain, liver and lung (Figure 9). AKAP7β mRNA expression is also detected in multiple tissues in rodent and human, though conserved expression between rodents and humans is only seen in the kidney, with the predominant AKAP7β expression site is the pancreas in humans (Figure 9).

## Discussion

In this study we have provided the first evolutionary analysis of an AKAP. The significance of PKA signaling has been shown throughout eukaryotic organisms. Here, we show the AKAP7 RI/RII binding domain appears in basal vertebrates, corresponding to a PKA-R subunit gene duplication event. In the molecular evolution of the AKAP7 gene, AKAP7 short splice variants appeared early in the vertebrate lineage and have remained highly conserved. In the AKAP7 long form splice variants, multiple nucleotide mutations and insertions-deletions have resulted in the concurrent loss of AKAP7γ and formation of AKAP7δ in rats. Specifically in rats, the replacement of AKAP7γ with AKAP7δ, as shown in this study, has likely lead to an over-statement of the significance of AKAP7δ in species other than rat [29,38,44,50,51]. Evolutionary pressures in the form of nucleotide insertions and mutations in the Human AKAP7δ coding sequence have likely driven preference for AKAP7γ production in humans. Hence, it is likely that AKAP7δ is of minimal significance in human AKAP7-mediated PKA signaling.

### Integration of the AKAP7-RI/RII binding domain and PKA-R Dimerization/Docking domain in vertebrate evolution

While AKAP7-like proteins were identified in invertebrate and chordate species, the appearance of the RI/RII binding domain (and formation of AKAP7 proteins) occurs in basal vertebrates (lamprey). The formation of AKAP7 proteins from AKAP7-like proteins corresponds with PKA-R gene duplication events where the PKA-RI gene duplicates to form PKA-RIα and PKA-RIβ, and PKA-RII forms PKA-RIIα and PKA-RIIβ. Evolution of the PKA-R subunits has been previously described [52]. Here we provide greater resolution of the timing of PKA-R gene duplications, and show that the amino acid sequences of PKA-R D/D domains are highly conserved, even prior to the gene duplication events. The relative fixation of PKA-R D/D sequences prior to and following the appearance of the AKAP7 RI/RII binding domain suggests that AKAP7 evolved subsequent to the increased presence of PKA (the result of PKA-R gene duplications). This is supported by the relatively rapid development of the AKAP7 RI/RII binding domain which is absent in chordates (ciona), but present in vertebrates (lamprey).

### Origin of AKAP7δ, the need for an AKAP7 long-form splice variant

As seen in our AKAP7 gene and splice variant phylogenetic analyses, AKAP7γ is the ancestral AKAP7 long form splice variant in mammals. Several nucleotide mutations identified in the murine lineage (as well as a basal primate) resulted in the loss of AKAP7γ through mis-sense mutations in the translational start codon. The most dramatic of these mutations occurs in rats, where the AKAP7γ initiation codon contains a successive transition and rare transversion that results in glycine replacing methionine. The results of these mutations have been confirmed in NCBI database searches, as rat AKAP7γ has been removed from the database (acc. #XM_001053676.1 has been removed), mouse AKAP7γ is truncated (acc. #NM_018747.4) and marmoset EST search results show AKAP7γ is not present whereas AKAP7δ is expressed (accession number XM_002746958.1). The combination of the AKAP7γ initiation codon loss combined with the concurrent absence of a downstream initiation codon utilized in mice (and present in the AKAP7 genes of several other species), likely yielded the evolutionary pressure for the incorporation of the AKAP7δ 5’ exon in rats.

In vitro analyses of mouse AKAP7γ, AKAP7δ and rat AKAP7δ show that both γ and δ splice variants are produced from the mouse AKAP7δ transcript, suggesting that AKAPγ is an internal translational start site product of AKAP7δ production. This is consistent with our AKAP7 gene analysis and provides a different model for AKAP7 long form splice variants than have been previously reported [29,53]. Our results also suggest that AKAP7γ is the major product of AKAP7δ transcription and translation. In rats, only a single protein product was identified from recombinant AKAP7δ expression which is consistent with gene and EST alignments showing the loss of AKAP7γ in rats. Findings from previous studies that have addressed AKAP7γ synthesis and function in rats [53,54] should be solely attributed to AKAP7δ as the AKAP7γ splice variant is absent in rats. The significant role of AKAP7δ in the renal aquaporin shuttle [29], and regulation of cardiac Ca^2+^ cycling [38] has led to a concerted effort to develop AKAP7δ peptidomimetics for use in treating human heart failure [44,50]. Results from this study show that AKAP7δ is the sole AKAP7 long form splice variant found in rats, and the significance of this splice variant is very likely over-stated. Combined with the identification of nucleotide insertions in the 5’ end of human AKAP7δ that would exclusively promote the production of AKAP7γ in all tissues, the evidence presented here indicates that the functional significance of AKAP7δ is minimal in species other than rats. In mice, where AKAP7γ appears to be the dominant product of AKAP7δ transcription and translation, cardiac AKAP7 immunlocalization is consistent with the SR indicating AKAP7γ largely replaces AKAP7δ at the SR in species other than rat and supports the possibility that AKAP7δ and AKAP7γ are functionally redundant in mice. Further studies are required to determine the functional consequences and distinction between AKAP7δ and AKAP7γ in mice and human skeletal muscle.

### Evolutionary pressure for replacing AKAP7δ with AKAP7γ

AKAP7δ has been identified as a vital component in cardiac Ca^2+^ handling at the sarcoplasmic reticulum in rats [38] and has been proposed as a pharmacological target in treating heart failure [44,50]. Our evolutionary analysis of the AKAP7 gene has shown that the origin of AKAP7δ was brought about through a series of nucleotide mutations in the AKAP7γ coding sequence in rats, hence likely leading to exaggeration of the functional significance of AKAP7δ. This is supported by *in vitro* analysis of mouse AKAP7δ, where AKAP7γ (through a likely internal translation initiation site) is the major product. AKAP7 immunoprecipitations from rat and mouse heart tissue also show AKAP7δ and an AKAP7δ variant in rat while in mouse heart AKAP7δ is a minor product and AKAP7γ is prominent. Furthermore, sequencing of human AKAP7δ (this study) showed the dominant presence (in multiple tissues) of a nucleotide sequence insertion immediately downstream of the AKAP7δ 5’ codon, resulting in a reading-frame shift that promotes the production of AKAP7γ. Therefore AKAPγ is the predominant, functional AKAP7 long form splice variant in humans and pharmacological efforts in designing AKAP7δ disruptor peptides should instead be directed at AKAPγ.

### AKAP7 short form splice variant evolution

Found only in the vertebrate lineage, AKAP7α is the ancestral AKAP7 splice variant and has conserved tissue expression in rodents and human heart, kidney, brain, liver and lung. AKAP7α has been extensively studied in the heart and brain, where this protein regulates PKA localization to (and phosphorylation of) ion channels such as the L-type Ca^2+^ channel [1,40,55,56] and epithelial Na^+^ channels [2,36,37,41]. Deletion of a specific region in the cardiac voltage-sensitive Ca^2+^ channel abolishes AKAP7 binding leading to cardiac hypertrophy and premature death in mice, and demonstrates the importance of AKAP7α mediated PKA signaling in adrenergic stimulation [57]. As seen in the expression results in this study, it is also likely that AKAP7α has a conserved role in ion channel regulation in kidney, liver and lung. AKAP7α amino-terminal amino acids that have been previously identified [1] in membrane targeting through myristolation (glycine at position 2; Figure 9) or palmitoylation (cysteins at positions 5 and 6; Figure 9) are absolutely conserved from zebrafish to humans. Furthermore, hydrophobic residues (leucine-4, phenylalanines-7 and 9, arginine-11) in the AKAP7α amino terminus which are likely to be critical for membrane localization are also conserved between zebrafish and humans. Given the high degree of sequence conservation in the AKAP7α amino terminus, it is likely this protein functions in membrane ion channel regulation throughout vertebrate species following the divergence of lamprey.

Development of the AKAP7β exon occurred in mammals (opossum), and while AKAP7β and α splice variants share the same amino terminus, the AKAP7β-specific exon confers a cellular localization distinct from the AKAP7α splice variant [46]. The functional significance of the AKAP7β splice variant in mammals remains unknown, though the sequence conservation in this protein is certainly suggestive of its importance. AKAP7β is primarily conserved in the kidney of rodents and human, and it seems likely that this splice variant has a conserved function in renal ion channel regulation. Importantly, in this study we have identified a novel AKAP7 long-form splice variant containing the β-exon. Further studies are required to address the unique membrane-localizing function of this AKAP7 protein region.

## Conclusions

Our results show that the formation of AKAP7 proteins occurred in basal vertebrate species, likely in response to the increased presence and distribution of intracellular PKA following PKA-R gene duplication events. Short form splice variants from AKAP7 genes have remained highly conserved across species, while long forms have undergone rapid evolutionary change. Independent mutations in the AKAP7 genes of rats, mice and marmoset have resulted in loss or truncation of AKAP7γ and a likely compensatory incorporation of AKAP7δ. These results suggest that studies of AKAP7δ are not necessarily comparable among rats, mice and humans. The specific targeting of human AKAP7δ in pharmaco-therapeutics should be undertaken with caution, as this splice variant is largely absent in humans.

## Methods

### Sequence data acquisition

Human and rodent AKAP7 splice variant sequences were obtained from NCBI and ENSEMBL (release 61) databases by searching for the gene “AKAP7” and AKAP7 transcripts. Annotated AKAP7 proteins were analyzed for conserved AKAP7 protein families (Pfams) using both the NCBI Conserved Domain Database (CDD) and Sanger Institute Protein Family Database ( http://pfam.sanger.ac.uk/) [58]. Conserved AKAP7 Pfams were identified as the AKAP7 Nuclear Localization Signal (AKAP7 NLS; Pfam 10469) and the AKAP7 RI/RII binding domain (pfam 10470). Human, rat and mouse AKAP7 Nucleotide and protein sequences obtained from NCBI ( http://www.ncbi.nlm.nih.gov) and ENSEMBL were used in BLASTP, TBLASTN and EST search algorithms to identify AKAP7 transcripts, proteins and genes in multiple species (listed below). Search results were verified to contain both AKAP7 Pfams (AKAP7 NLS and AKAP7 RI/RII). Sequences obtained from these searches that contained only the AKAP7 NLS Pfam were classified as “AKAP7-like proteins. Multiple species spanning the vertebrate and invertebrate lineages were used for this study, specifically: primates-*Homo sapiens (H. sap)Pan trogylodytes (P.tro)Macaque mulatta (M. mul) Callithrix jacchus (C. jac)*, rodents-*Mus musculus (M. mus)Rattus norvegicus (Rnor)Oryctolagus cuniculus (O. cun)*; laurasiatheria- *Canis familiaris (C. fam)Equus caballus (E. cab)Bos Taurus (B. tau)Sus scrofa (S. scr)*, metatheria- *Monodelphis domestica (M. dom)*, reptiles- *Anolis carolinensis (A. car)*, amphibians- *Xenopus tropicalis (X. tro)*, teleosts- *Danio rerio (D. rer), Takifugu rubripes (T. rub), Oryzias latipes (O. lat), Gasterosteus aculeatus (G. acu)*; vertebrates- *Petromyzon marinus* (*Pmar*); chordates- *Ciona intestinalis (C. int)*.

### Development of PKA-R and AKAP7 interaction

To determine the relationship between AKAP7 RI/RII binding domain and PKA-R gene evolution, we constructed a PKA-R phylogenetic tree from the PKA-R cAMP binding domains, and identified AKAP7 RI/RII binding domain presence/absence for each species used in the PKA-R phylogenetic tree. PKA-R translated amino acid sequences were aligned using the Clustal function of Mega v5 [59]. PKA-R subunit protein relationships were determined using the algorithm of Mr. Bayes 3.1.2. Mr. Bayes analysis was run under a fixed rate Dayhoff model. The tree is a representative of 4 independent runs, of four million generations each. The tree was rooted with *Drosophila melanogaster*. Pictoral representation of AKAP7 RI/RII presence/absence is shown next to the PKA-R clades that contain this motif.

### EST to Gene alignments

To determine AKAP7 splice variant exon positions and gene structures, the AKAP7 genes of human, rat, dog, opossum, zebrafish, lamprey, and ciona were analyzed using GENESCAN [60] and Promoter 2.0 Prediction Server [61] FPROM (Softberry, Inc., Mt Kisco, NY) programs. AKAP7 splice variant exon positions and splice junctions were verified by using NCBI two-sequence BLAST (bl2seq) and manually aligning cDNA sequences to the genomic sequences using Mega v5 [59].

### Animals

All experiments involving animal subjects were carried out with the approval of the University of Illinois-Chicago (UIC) and Jesse Brown VA Medical Center (JBVAMC) Institutional Animal Care and Use Committees (IACUC). Adult male rats and mice were used for cardiac and renal tissue preparations in DNA sequencing, western blotting and immunofluorescence applications.

### AKAP7 Splice variant detection and sequencing

Human multiple tissue cDNA (MTC panel I, CloneTech, CA) was used in PCR amplification and sequencing applications. Rat and mouse total RNA were prepared using Trizol reagent (Invitrogen). Five micrograms of total RNA was used in first-strand cDNA synthesis using SuperScript III Reverse Transcriptase (Invitrogen) primed with random hexamers. PCR was performed using KOD Hot Start DNA polymerase (EMD Biosciences). Primers used for the amplification of human, mouse and rat AKAP7α, β, and δ splice variants are listed in Table 1. PCR products were extracted from agarose gels and cloned into the pGEM-T vector system (Promega) and sequenced using a capillary sequencer (Applied Biosystems).

**Table 1:** Primers used in PCR amplifications and Sequencing

**Name**	** Sequence 5’ to 3’**
Human AKAP7δ For	ATGGAGCGCCCCGAAGCGGGAGGAATTAAT
Human pan AKAP7 Rev	TCATTTCCTGTTGTTCTCATTGTCATTGCCATTCTGATCA
Human AKAP7α For	ATGGGCCAGCTTTGCTGCTTTCCTTTCTCAAG
Human AKAP7β For	GAGTTGGAAAGCTCGTCCTCTGCAGTCCTAC
Human AKAP7δ-indel set 1 For	ATGGAGCGCCCCGAAGCGG
Human AKAP7δ-indel set 1 Rev	TTTCCTCTTCTTTACTTGATCACTCTTGACCCATTCATT
Human AKAP7δ-indel set 2 For	TTCATAGAAGAACTCCTCCAGGGAAAACATTTGACTTTG
Human AKAP7δ-indel set 2 Rev	GAAGATTCACAGTGATAATAACCATTACTTTGTTTT
Human AKAP7δ-indel set 3 For	CCTGGTAGGAGAGAGCAGAAGTTTTAAACCTCATTTGAC
Human AKAP7δ-indel set 3 Rev	TCCACCAGCCTCTTACTGAGCCTTACTAGTTC
Mouse AKAP7δ For	CACCATGGAGCGCCCCGCCGT
Mouse pan AKAP7 Rev	CTTCCGGTTGTTATCACTGCCATCACCATTCCGAT
Mouse AKAP7γ For	CACCATGCCGTTTGCTGCTGTAGATATTCAAGATGA
Mouse AKAP7α For	ATGGGCCAGCTTTGCTGCTTCCCTTTCG
Mouse AKAP7β For	GAGTTGGAAAATCCATCTTCTGCAGACCTACAGAG
Mouse AKAP7γ-short Rev	TTAGTGCCCAGGGTGGAGCTTTGGAGCAGT
Rat AKAP7δ For	CACCATGGAGCGCCCCGCCGCGGGA
Rat pan AKAP7 Rev	CTTCCGGTTGTTATCACTGCCATCGCCATTCC
Rat AKAP7α For	ATGGGCCAGCTTTGCTGCTTCCCC
Rat AKAP7β For	CAGTTGGAAAGTCCATCTTCTGCAATCCTACAGAGAT

### Transfections

Mouse AKAP7δ, AKAP7γ and rat AKAP7δ transcripts were PCR amplified from heart or kidney cDNA templates and cloned into a pcDNA3.1/V5-His expression vector (Invitrogen) to produce V5-tagged proteins. For transient transfection purposes, 2x10^5^ HEK293 cells (American Tissue Culture Collection) were grown on 6-well plates for 24 h in Dulbecco's Modified Eagle Medium, 10 % Fetal Bovine Serum (FBS) (BioWhitaker), 100 U/mL penicillin and 50 μg/mL streptomycin. Cell transfections were performed by using Lipofectamine 2000 (Invitrogen Life Technologies) according to the manufacturer’s instructions. Cells were transfected with mouse AKAP7δ, mouse AKAP7γ, or rat AKAP7δ. Transfected and mock-transfected cells were harvested 48 hours after transfection and washed with PBS followed by sonication in deionized urea-thiourea-chaps buffer (UTC; 8 M urea, 2 M Thiourea, 2 % chaps) with protease and phosphatases inhibitors (Pierce). Supernatants were then used in 2D western applications.

### Immunoprecipitations

Immediately following removal, rat and mouse hearts were fast-frozen in liquid nitrogen and dounce homogenized at 4°C in 50 mmol/L of Tris–HCl (pH 7.4), 150 mmol/L of NaCl, 1 mmol/L of EDTA, 0.25 % sodium deoxycholate, 1 % triton X-100 and 1X HALT protease and phosphatases inhibitor cocktails (Pierce). The homogenate was centrifuged at 10,000X RCF for 15 minutes at 4°C, the supernatant was collected and protein concentrations were determined using the Dc Protein Assay (BioRad). Five hundred micrograms of the heart lysates were incubated in 500 μl lysis buffer containing 1 μg of rabbit polyclonal AKAP7 antibody with slow rotation on a rocker overnight at 4°C. Negative control immunoprecipitations were carried out using rabbit IgG (pre-immune serum) instead of the AKAP7 antibody. Lysate-immunoglobulin samples were then incubated with 50 μl of protein A/G agarose beads (EMD) for 2 hours under constant rotation. The samples were then centrifuged, supernatants were removed and agarose bead pellets were successively washed in PBS containing 1.00 %, 0.5 %, and 0.05 % Tween-20. The beads were resuspended in deionized UTC buffer with protease and phosphatases inhibitors, mixed and incubated at room temperature for 10 minutes. Following centrifugation, the supernatants were used in 2D Western applications.

### Two-dimensional western analysis

Samples from transfections and heart lysate immunoprecipitations were centrifuged for 10 minutes at 10,000 RCF and the protein content of the supernatants was estimated using the RC/DC protein assay (BioRad). Fifty micrograms of total protein was isoelectrically focused (IEF) using 7 cm isolated pH gradient (IPG, pH 3–10 non-linear gradient) strips on an IEF cell (BioRad). Second-dimensional separations were performed on 4-15 % gradient tris-glycine SDS-PAGE gels. Proteins were transferred to PVDF membranes for western analysis. To detect recombinant AKAP7 proteins from transfections, membranes were probed using an anti-V5 HRP-conjugated antibody (Invitrogen) at a 1:5000 dilution. Proteins from AKAP7 immunoprecipitations were detected with a protein-A HRP conjugate. In negative control immunoprecipitations, rabbit IgG was used in place of the AKAP7 antibody. Western blot images were taken on a Kodak image station 4000R pro.

### Immunofluorescence microscopy

Affinity-purified rabbit polyclonal pan-AKAP7 antisera was produced by Genscript corp. (Piscataway, NJ) for use in immunoprecipitations and immunofluorescence applications in rat and mouse hearts. The epitope used for AKAP7 antisera production was “LVRLSKRLVENAVC” which is common to rodent and human AKAP7 splice variants. Following removal, rat and mouse hearts were fixed overnight with 10% formalin in 0.1 M phosphate buffer (pH 7.2). Tissues were dehydrated in graded ethanol solutions and embedded in paraffin. Nonspecific staining was blocked by incubation with 1.5 % normal goat serum in PBS for 1 hr at room temperature in a humidified atmosphere. The sections were incubated with the anti-AKAP7 antibody or normal rabbit serum (for negative control) overnight at 4 C. After three washes of 10 min each in PBS-T, the sections were incubated with Alexa fluor 488-conjugated anti-rabbit secondary antibody (Invitrogen) for 2 hours at room temperature and washed three times. Nuclei were counterstained with Dapi.

## Abbreviations

AKAP: A-Kinase Anchoring Protein; PKA-R: PKA regulatory subunit; PKA-R D/D: PKA regulatory subunit dimerization/docking domain; SERCA: Sarcoplasmic-Endoplasmic Reticulum Calcium ATPase; PLN: Phospholamban; ORF: Open Reading Frame..

## Competing interests

The authors declare that they have no competing interests

## Authors’ contributions

KRJ conceived the study, designed the experiments, obtained data, interpreted data, and drafted the manuscript. JNJ constructed the PKA-R phylogenetic tree and edited the manuscript. GKC provided technical advice and edited the manuscript. RSD edited the manuscript and supported study. All authors have read and approved the manuscript for publication.

## Supplementary Material

Additional file 1 **Figure S1. AKAP7 Splice Variant Evolution.** EST classification and EST to gene matching shows the differential incorporation of AKAP7 splice variants throughout evolution. A cladogram was constructed using the NCBI taxonomy common tree tool and matches known species phylogenetic relationships. The accession numbers from NCBI or Ensemble databases and corresponding exon-intron structures in each species (determined by EST to gene matching) for the AKAP7 splice variants is shown in each column. A splice variant is indicated as “not present” if the exon(s) for that splice variant are absent from the AKAP7 gene. A splice variant is indicated as “not detected” if the necessary exons are identified in the AKAP7 gene but no EST was identified. Splice variants with a non-orthologous AKAP7γ exon-1 are listed as AKAP7γ-like. Click here for file

Additional file 2 **Figure S2. Nucleotide and Amino acid alignments of human, mouse and rat AKAP7δ.** (A) Human and mouse AKAP7δ splice variants were sequenced and aligned to the previously reported rat AKAP7δ nucleotide sequence. Predicted mutations in the AKAP7γ initiation codon are confirmed, and indicated by an (*). There is 78-89 % sequence identity between rat, mouse, and human AKAP7δ nucleotide sequence. The least similar region is found between nucleotides 180 and 200, (B) translated amino acid sequences were aligned using ClustalX2. Rat and mouse AKAP7δ contain an “RGD” domain between amino acids 70 and 75, this domain is absent from the human sequence. Click here for file

Additional file 3 **Figure S3. Splice variants of Rat AKAP7δ.** (A) PCR amplification of AKAP7δ from rat heart cDNA yielded two smaller splice variants in addition to the native form. (B) Rat AKAP7δ variant-1 has an exon-7 deletion, whereas AKAP7δ variant-2 consists of exons 1, 2 and 8, while exons 3–7 are deleted. Rat AKAP7δ variant-1 contains the AKAP7 NLS (dotted underline) but not the AKAP7 RI/RII binding domain. Rat AKAP7δ variant-2 lacks the AKAP7 NLS but contains the AKAP7 RI/RII binding domain (box). Click here for file

Additional file 4 **Figure S4. Mouse AKAP7γ splice variant.** A short splice variant consisting of AKAP7γ exons 1 and 2, and a unique 3’ exon was identified in mouse ESTs. (A) This truncated splice variant contains the AKAP7 NLS (dotted box), but lacks the AKAP7 RI/RII binding domain. A model was constructed (B) to show the relative positions of constituent exons. (C) The mouse AKAP7γ short variant is not widely expressed, and is detected only in the spleen. Click here for file

Additional file 5 **Figure S5. Nucleotide and amino acid alignments of AKAP7 short form splice variants.** (A) The amino terminus of AKAP7α and AKAP7β was aligned using clustalX2. Amino acid sequences are highly conserved from zebrafish to humans. Residues that have been previously identified as lipid-modified [1] are indicated by (‡) and hydrophobic residues are indicated with down-arrows (↓). (B) The AKAP7β-specific region is identified in species from pig to humans, with several highly conserved residues which are indicated with (*). Click here for file
